# The impact of gout on patient’s lives: a study of African-American and Caucasian men and women with gout

**DOI:** 10.1186/ar4589

**Published:** 2014-06-24

**Authors:** Jasvinder A Singh

**Affiliations:** 1 Medicine Service, Birmingham VA Medical Center, Birmingham, AL, UK; 2 Department of Medicine at School of Medicine and Division of Epidemiology at School of Public Health, University of Alabama, Faculty Office Tower 805B, 510 20th Street S, Birmingham, AL 35294, UK; 3 Department of Orthopedic Surgery, Mayo Clinic College of Medicine, Rochester, MN, USA

## Abstract

**Introduction:**

The aim of this study was to examine the impact of gout on quality of life (QOL) and study differences by gender and race.

**Methods:**

Ten race- and sex-stratified nominal groups were conducted, oversampling for African-Americans and women with gout. Patients presented, discussed, combined and rank-ordered their concerns.

**Results:**

A total of 62 patients with mean age 65.1 years, 60% men, 64% African-American, participated in 10 nominal groups: African-American men (n = 23; 3 groups); African-American women (n = 18; 3 groups); Caucasian men (n = 15; 3 groups); and Caucasian women (n = 6; 1 group). The most frequently cited high-ranked concerns among the ten nominal groups were: (1) effect of gout flare on daily activities (n = 10 groups); (2) work disability (n = 8 groups); (3) severe pain (n = 8 groups); (4) joint swelling and tenderness (n = 6 groups); (5) food restrictions (n = 6 groups); (6) medication related issues (n = 6 groups); (7) dependency on family and others (n = 5 groups); (8) emotional Impact (n = 5 groups); (9) interference with sexual function (n = 4 groups); (10) difficulty with shoes (n = 4 groups); and (11) sleep disruption (n = 4 groups). Compared with men, women ranked the following concerns high more often: problems with shoes (n = 4 versus n = 0 groups); dependency (n = 3 versus n = 2 groups); and joint/limb deformity (n = 2 versus n = 0 group). Compared with Caucasians, African-Americans ranked the following concerns high more often: dietary restrictions (n = 6 versus n = 0 groups); severe pain (n = 6 versus n = 2 groups); gout bringing the day to a “halt” (n = 2 versus n = 0 group); effect on emotional health (n = 4 versus n = 1 groups); and the need for canes/crutches during flares (n = 2 versus n = 0 group).

**Conclusions:**

Gout has a significant impact on a patient’s QOL. Important differences in the impact of gout by gender and race were noted.

## Introduction

Gout is the most common type of inflammatory arthritis in adults. Gout affects 8.3 million Americans [1] and 5% of the US veterans [2]. It leads to frequent emergency room visits [3] and costs 20 billion dollars annually [4]. Despite the availability of effective treatments, patients with gout do not receive good-quality gout care [2,5-10] as recommended by gout treatment guidelines [11,12]. Quality gaps are worse in racial minorities and women [13]. Significantly lower rates of urate-lowering therapy (ULT) prescriptions and ULT adherence are seen in African Americans [14,15] compared to Caucasians and in women compared to men [7,16]. Patient perceptions can impact gout outcomes. An example is that negative or pessimistic patient views about gout are associated with poorly controlled gout, low adherence to ULT and higher disability [17]. This implies that better insights into patient experience and perception of gout are needed, especially in African Americans.

Gout has a significant negative effect on patient’s quality of life (QOL) [18-21] and effective treatment improves QOL [22,23]. Therefore, it is critical to understand the specific impact of gout on patients’ lives. To date, qualitative research in gout has focused on examining patients’ knowledge related to gout, patterns of treatment and the barriers to treatment adherence [24-26]. There are at least three knowledge gaps related to the effect of gout on QOL: (1) qualitative studies have been done primarily in Caucasian men with gout; none included African Americans [24-26]; (2) only 11 of the 46 gout patients in all previous studies combined were women [24,25], indicating the need for more research into women with gout; and (3) most studies have assessed multiple aspects of gout, including treatment, knowledge, disease monitoring, outcomes and patient and clinician perspective, often in a setting of a single study [24-26]. Thus, there is a lack of in-depth studies assessing the impact of gout on QOL.

The lack of research in African Americans with gout, a racial group with higher disease prevalence and burden of gout than Caucasians (5% versus 4% in the US), is concerning [1]. Although gout is not as common as it is in men, the prevalence is 2% in US adult women [1], twice that of rheumatoid arthritis at 1% [27]. A literature search using the same terms (quality of life, women) found eight publications in gout (none with original data, none relevant) versus 213 publications in rheumatoid arthritis, indicating the lack of recognition of the impact of gout on women.

Corbin and Strauss proposed the trajectory model to understand the impact of illness further refined by Charmaz and others. The model includes three components - body, biographical time (explicit narrative that gives meaning and purpose to a person’s life) and conceptions of self (constructs of self-image and identity, such as role identity, social identity et cetera), that is, the BBC chain [28,29]. The model indicates that only when body, biographical time, and conceptions of self are in balance, interactively stabilizing and reinforcing one another, does one enjoy a sense of health and wellbeing [28-30]. When this chain is destabilized, one has the sense of being ill. A chronic disease may lead to progressive loss of self from body failures, failed performances and social isolation [31]. The study question is based on this theoretical model and the study findings were mapped to this model. The objectives of this study were to: 1) assess the impact of gout on patients’ QOL; and (2) explore gender and race differences in the impact of gout on patients’ QOL. We oversampled for African Americans and women to get a diverse perspective.

## Methods

### Patients

The Institutional Review Board at the University of Alabama at Birmingham approved the study. Patients with at least one outpatient visit between January 2011 to September 2012 to our outpatient clinic, with an International Classification of Diseases, ninth revision, common modification (ICD-9-CM) code for gout, that is, 274.xx, were eligible and invited for study participation on the phone. Consecutive patients were selected. African Americans and women were oversampled, due to the lack of qualitative research in these groups. Patients were invited for 1.0 to 1.5 hour sessions. Study participants received free parking, refreshments during the session and a $30 check. Patients provided consent prior to study initiation.

### Nominal group technique (NGT) sessions and analyses

Patient nominal groups were conducted to understand the impact of gout on the patient’s QOL and evaluate gender and racial differences. NGT is a variant on traditional focus group with methods aimed at developing an inclusive list of issues related to a specific question, then soliciting feedback on the relative importance of these lists through rank-ordering procedures [32,33]. NGT has been used successfully in a variety of medical conditions with assessments from experts, professional caregivers and patient groups [33-39]. NGT taps the experiences, skills, or feelings of the participants by promoting an even participation. Thus, the data generated usually provide a more valid representation of the implicit views of the group than would be achieved with a focus group format. Generally, two to three groups are considered enough to reach saturation.

Given that QOL is not a lay-person concept, careful consideration was given as to how to ask the question about how gout affects patients’ QOL. After several discussions with pyschometricians, physicians, and patients with gout in the clinical practice, the question was finalized as, ‘How does gout affect your life?ʼ The nominal groups were stratified by gender and race/ethnicity. The nominal group leader (JAS), an epidemiologist experienced in conducting nominal groups [40,41], conducted the nominal group sessions. Research assistants (BA, AO or AB), provided administrative support, recorded the discussions and took notes. Each session started with introductions followed by asking the participants if the main question was clear and if they had any questions prior to starting the group meeting. All queries were answered before the start of each NGT session. The main question was written on flipchart and also printed on top of a blank sheet of paper provided to each participant.

The NGT process consisted of the following discrete steps that were completed in 60 to 70 minutes, as planned: (1) participants briefly and independently generated as many words or short phrases as possible in response to the question on a sheet of paper with the question (10 to 15 minutes); (2) participants nominated a single response in a round-robin fashion, which was recorded on a flip chart in large letters visible to the group participants by the group leader; responses are nominated until all identified by any group member were recorded; (3) once the list was completed, participants discussed and elaborated each response and where appropriate combined responses deemed alike; and (4) all participants were asked to identify and rank-order the five responses deemed most important from 1 to 5, with 5 being the highest score on the index cards. All scores were totaled. A rank-order was created based on the total scores, with the highest score corresponding to the top rank.

Rank-ordered results from each nominal group were created. Similar to the within-group process, responses were analyzed based on the number of nominal groups identifying the concept with high relative rank-ordering. A general consensus rank-ordered list was created. Transcriptions were examined to identify all statements made relative to each response (discussions directly connected, solutions generated, and any point where the response was brought up subsequently) and led to the creation of a comprehensive list of statements. To ensure that nominal groups were performed until saturation, as groups were completed, responses were compared to determine overlap. When significant overlap was noted, saturation was confirmed.

## Results

Ten nominal groups were conducted with 62 gout patients: 60% were men and 40% women, 64% African American and 36% Caucasian. The mean age was 65.1 years (standard deviation, 10.8). Three nominal groups each were conducted with African American men, African American women, and Caucasian men and one nominal group with Caucasian women. Saturation of themes was achieved by the third group in each group. The mean age (standard deviation) of the four cohorts that participated in nominal groups were: African American men, 61.8 (12.4); African American women, 68.9 (10.4); Caucasian men, 63.8 (12.6); and Caucasian women, 61.3 (2.3).

### Overall impact of gout on quality of life

Several themes emerged. The top seven (or so) themes/concerns from each group are listed in Tables 1 and 2. In the section below, we list the top seven concerns nominated by more than one nominal group. These concerns are categorized as identity-relevant performances with or without loss of self versus body failures, although some themes may fall into more than one category.

**Table 1:** Top ranked seven concerns* among African American and Caucasian men

	**Quotes**	**Votes, number**
African American men (n = 5)		
Impact of flare on social life	‘Affects my social life - Going to dinner, church, movies, etcʼ	10
	‘I don’t want to be around anybody during the attackʼ	
Pain during and after the flare and its impact	‘…(flare causes)…extreme painʼ	9
	‘Crying while I am in painʼ	
	‘It makes me emotional and mean when I have an attackʼ	
Limitation of recreational activities	‘Things that I like are taken away (music)ʼ	6
	‘I can’t go (places…) with my brothersʼ	
	‘Affects my hobbies - fishing, hunting, biking, playing music, riding motorcycle, runningʼ	
Work productivity	‘Hopefully your boss understandsʼ	6
	‘Relationships are hard at work tooʼ	
	‘Caused me to take off from (or ability to do) my job.ʼ	
Unpredictability of flare that brings a normal day to a halt	‘Brings normal day to a haltʼ	5
	‘Stopping the desire to do anythingʼ	
	‘Comes unexpectedʼ	
	‘Don’t even want someone breathing around meʼ	
Dietary restrictions and modification	‘What do different foods contribute to gout?ʼ	5
	‘Staying away from fish, shellfish, steaks, & peasʼ	
	‘Affects foods I can enjoyʼ	
	‘Always have to watch what I eat - staying disciplinedʼ	
Feeling of loss of youth	‘I was diagnosed in my 20s… feel old…	5
	‘Causes me to feel “old” before my timeʼ	
African American men (n = 9)		
Severe pain and disability during an attack	‘When I have an attack it shuts down my whole life. I can't function. I will stay in the bed three to four days while I wait for it to passʼ	36
	‘Have to have wheel chair close to me during an attackʼ	
	‘I have to keep my crutches by the bed so that I can get to the bathroomʼ	
	‘You will do anything to get reliefʼ	
	‘I miss going to church and other functionsʼ	
Swelling and tenderness in acute flare	‘My toe ankle and knee they really swell up.ʼ	16
	‘…tenderness in the acute phaseʼ	
Affects sleep	‘Limits my ability to have a good night sleepʼ	16
	‘You just can't sleepʼ	
Dietary restrictions and modification	‘Affects what I can eatʼ	14
	‘Can’t have a few drinks without a flareʼ	
	‘Can't eat red meat, even though I love itʼ	
Need for daily medication	‘…have got to take medication every dayʼ	11
	‘I have to take additional medications to stay wellʼ	
	‘It’s inconvenientʼ	
	‘It’s just another drug when you add that to our other medicationsʼ	
	‘I also have to take a pain medicationʼ	
At-home productivity and dependency on family members and others	‘It disables me. I need help with my socks or just walking around the houseʼ	9
	‘I can't use lawn mowerʼ	
	‘Need help from family and othersʼ	
	‘During a flare up I need help just to get me to the doctorʼ	
	‘One time the fire department had to find a way to get me out of the bed, into a wheelchair, into the back of a truck then to the hospital… It took a whole lot of help to get to the doctor that dayʼ	
	‘I can't do anything someone will have to bring me food, help me in and out of the bed; hop to the bathroom during an attackʼ	
Affect on family life and sexual function	‘I can’t go to my grandson’s gameʼ	9
	‘I can't do the things I like to do with my wifeʼ	
	‘My relationships are affected… I don't want anyone around me to touch meʼ	
African-American men (n = 9)		
Dietary restrictions and modification	‘Made me change my dietʼ	24
	‘I had to change the food that I ateʼ	
	‘I can’t eat seasoned food; I did not like that at allʼ	
	‘No seafood, shell fish, red meat and fried foodsʼ	
	‘Watch what you drink - no alcoholʼ	
	‘Can’t have sodas or drink orange juiceʼ	
	‘Can’t have peanuts, pecans and tomatoesʼ	
Problem with daily activities	‘Hard to move aroundʼ	18
	‘Very hard to move around during a flare. It stays with me for a while, several daysʼ	
	‘Could not stand and it would hurt if the cover was touching itʼ	
	‘Have to be careful what you can doʼ	
	‘No activity outsideʼ	
	‘It's aggravating, not being able to do anythingʼ	
	‘All you want is the pain to go awayʼ	
	‘Gout affects my drivingʼ	
	‘During the flare there is a problem going to the restroomʼ	
	‘I couldn't drive my truck if my gout was flaring. I had to learn to shift with my left foot. My diet, activities and everything had to change because I could not do it like I used toʼ	
Sexual difficulties	‘Problem with having sex during the flare. When you are in pain… the love goesʼ	14
	‘You don't want to do anything when you are in pain. Our old lady doesn't understandʼ	
Problem with shoes/footwear	‘I wore a wider shoeʼ	14
	‘I had to get shoes 2 sizes largerʼ	
	‘Needed hard leather shoes and loose fitting shoesʼ	
	‘Get out of the fancy shoes and wear some soft shoesʼ	
	‘I wear sandalsʼ	
Joint swelling	‘Affects my joints - I have swellingʼ	12
	‘I will have small swelling in my feetʼ	
Life-changing impact	‘Made my life change in a lot of waysʼ	9
	‘My whole life changed. I had to accept a lot of things that I did not want toʼ	
	‘Pain made me change in a lot of ways with my friends family and the people that I am normally around. It will have an impact on the way your carry yourselfʼ	
Severe pain during flare	‘Can’t have anything touching it a sheet, clothes… nothing.ʼ	9
	‘…when it flares up you can't sleepʼ	
	‘I have to sleep a certain way to avoid painʼ	
	‘I have to sleep in a chairʼ	
Emotional impact	‘Can’t play with painʼ	7
	‘Makes me irritatedʼ	
Caucasian men (n = 5)		
Problem with daily activities and at-home productivity	‘Gout affects my physical abilitiesʼ	17
	‘I can't garden or do yard work anymore. Can't do vegetable gardeningʼ	
	‘Work around the house is impossible when I have a harsh gout attackʼ	
	‘…(during flare)… My sink is full of dishesʼ	
	‘Standing is a devil (during flare).ʼ	
	‘My inability to walk and move around totally limits my life (to go to the bathroom, eat do anything)ʼ	
Impact on family life and sexual function	‘Affects your sex lifeʼ	10
	‘My life with family has sufferedʼ	
Work productivity	‘My work output suffersʼ	8
	‘I get paid on commission. My average has dropped over the years due to goutʼ	
	‘Affects my job. A limitation to the jobʼ	
	‘The medication (narcotics) has made it difficult to workʼ	
Long-term medication side-effects	‘Affects your personality usually due to the medication that you are taking (steroids)ʼ	7
	‘I worry about the long term affects the drugs have on my healthʼ	
	‘The side affects that I have or could get from the medication or the diseaseʼ	
	‘My joints are deteriorated due to me continuing to move during a flare upʼ	
	‘Because I have one kidney my pain reliving drugs are even more limited“	
Impact on comorbidity management	‘How it (gout) interacts with my other health issuesʼ	6
	‘Raises blood pressureʼ	
	‘That's my problem - I do not know how it is affecting my other problems. I ask my doctor all the timeʼ	
Dependency on family members	‘I don't like to be waited on… they should not have to bear that burdenʼ	5
	‘I have to have help when I am having a severe attackʼ	
	‘Concern over being dependent on my wife and family because of my limitationsʼ	
Makes traveling very tricky		5
Caucasian men (n = 7)		
Intense pain	‘Gout leads to severe painʼ	24
	‘Attack in the unusual placesʼ	
	‘Unable to live the active life tat I liveʼ	
Gout stops certain activities	‘Limited movementʼ	18
	‘….couldn’t walkʼ	
	‘Inability to exercise, have family interactionsʼ	
	‘..(had to) reduce daily activitiesʼ	
	‘Limitation of mobilityʼ	
Not predictable	‘Don’t you wish you knewʼ	18
	‘Can not plan activities in town and out of townʼ	
	‘Out of town, can not get medical attention quick for an attackʼ	
Need for daily medication	‘Take medication everyday and buy itʼ	12
	‘If you travel you are stuck with themʼ	
	‘…(problem) having to take daily medicationsʼ	
Expenses of medications and doctor visits	‘I have group health insurance and my condition has impact on othersʼ	8
	‘Limitation of disposable incomeʼ	
	‘….emergency room visits are expensiveʼ	
Lack of healthiness	‘….contributes to the sense of lack of healthinessʼ	6
Possible drug interactions	‘Lot of unexplained symptoms, wondering if they might be due to side effects of allopurinolʼ	6
	‘We are getting older and need to worry about theseʼ	
	‘What about it’s effect on medications for other diseasesʼ	
Caucasian men (n = 3)		
Severe pain	‘Pain was so much, I could hardly walkʼ	11
	‘I broke my leg an year ago, I swear gout hurt worse than my fractureʼ	
	‘I broke my ankle, not even comparable to acute gout attack (gout was worse)ʼ	
	‘Pain from gout was worse than neck fusion symptomsʼ	
	‘I can feel the pain with every heartbeatʼ	
Physical limitation	‘Limits my ability to play sports, family events and excursions, vacation, home improvement and choresʼ	12
	‘Limits sporting events during the attackʼ	
	‘Could hike, if it weren’t thereʼ	
	‘Has long-term impact on hobbiesʼ	
	‘I had to give up sports I played my whole lifeʼ	
	‘Had to so “no” a lotʼ	
	‘Had an attack during vacation. I couldn’t do things and make me think of polioʼ	
Emotional impact	‘Puts emotional strain in relationships, marriage and familyʼ	7
	‘People have heard about it, but don’t know what it isʼ	
	‘It led to my divorceʼ	
	My wife said in her journal “… seems that he wants to be in pain to avoid doing things with meʼ	
	‘My family and wife know very well what gout isʼ	
Limits work ability	‘Limits my ability to workʼ	6
	‘Delay, reschedule or ask coworkers (ask for help)ʼ	
	‘Traveling is difficultʼ	
	‘Can’t driveʼ	
	‘….had to get cherry juice and spend a night in a hotelʼ	
Need to take daily medication	‘Have to take medication dailyʼ	4
	‘Not easy to fill and refill, especially when travelingʼ	
	‘I don’t like taking medications every dayʼ	
	‘Allopurinol if a miracle drugʼ	
Awareness of joint disease	‘Constantly cautious of joint sensitivityʼ	4
	‘Anytime I feel something it constantly reminds me of my attacksʼ	
Finding fellow people with gout	‘Bond with people who know how it feelsʼ	1
	‘Becoming part of social interactionʼ	
	‘Every friend of mine knows I have goutʼ	

*Since some concerns were ranked equally by a group and tied for a rank, some groups have more than seven concerns.

**Table 2:** Top ranked seven concerns* for African-American and Caucasian women

	**Quotes**	**Votes, number**
African-American women (n = 3)		
Constant pain	‘Having to continue day to day tasks even with constant painʼ	9
Interactions with other medications	‘I have high blood pressure and my gout medication would raise my blood pressure. The same issue with my diabetes medicationʼ	9
	‘Medical doctors say don't take something that the rheumatologist said would help my gout because it would mess up other thingsʼ	
Concern and confusion regarding dietary restrictions	‘You get different lists when you Google this question. It says that everyone has to find what starts the attack for themʼ	7
	‘Red meat and some fish…ʼ Any fish? ‘…(avoid) Dark green leafy vegetables.ʼ	
	‘I don't notice foodʼ	
	‘Unsure of what and what not to eatʼ	
Joint swelling with discomfort and deformity	‘This is a social aspect because I get self conscious because my feet are so swollen.ʼ	5
	‘Residual swelling in joints constantly in feet, unable to wear my shoesʼ	
	‘My hand swells. I can't put my rings on some morningsʼ	
	‘Supposed to have a brace on my hand for the swelling. I may have to have surgery on the handʼ	
	‘(Swelling)… on top of my foot or my big toeʼ	
	‘Swelling in my shins and everywhere!!!ʼ	
Severe pain	‘As soon as you take your foot of the bedʼ	5
	‘Can't even have the cover touching my feet.ʼ	
	‘Can even just lay there and still have painʼ	
Concern about taking too many pills	‘I am taking more pills than I would like to take to get rid of the pain and get any kind of sleepʼ	3
Problem with daily activities, at-home productivity and dependency on family during gout flares	‘When I have an attack, I can't walk; can’t take care of my family, my home or myselfʼ	2
	‘I can’t get to bathroom, cook wash, or go to the storeʼ	
	‘I didn't care if anything was done because the pain was so severe. I also didn't know what it was. I couldn't walk they thought it could be a stress fracture until the blood work was reviewed. You are so concerned with pain you can't do anything. I would literally scream every time I would put my feet down. The first attack was in my shoulder I could not move it. I was given morphine and it was still hurting	
	‘The prednisone makes me hold fluid. I have asked to be taken off of it; leaving me taking medicine I would not like to takeʼ	
	‘Can’t walk it's so painful; I have to use crutches hop on one leg or crawlʼ	
	‘During an extreme onset unable to walk at allʼ	
	‘Can’t clean the houseʼ	
	‘Family can't take care of my grandchildren properly. I have an ADHD child I have to keep up withʼ	
African-American women (n = 8)		
Extreme pain	‘Hurts when I lay on my side at nightʼ	26
	‘Sleepless nights when I get an attackʼ	
	‘It’s in my foot - it's hard to walk - I can't put on any shoes or touch it like a sheet or anything like thatʼ	
	‘I can't stand for nobody to touch it… my feet when I have itʼ	
	‘When it flares it's very painful. It causes me not to move itʼ	
	‘My feet feel tender it makes me walk different. Like I'm hoppingʼ	
	‘Affects my life very very bad… It's sorer than anything else. I cannot walk. All I can do is hop to the bedʼ	
	‘Sudden sharp pains in legs, thighs, and foot during and between attacksʼ	
	‘I just woke up and couldn’t have anything touching meʼ	
Swelling and heat in the area of attack	‘I have hurting and swelling in my right kneeʼ	21
	‘It's in my hands and knee right now they are treating it with cortisoneʼ	
	‘Causes swellingʼ	
	‘Affected area is hotʼ	
Constant pain	‘I am in constant painʼ	15
	‘It never stops hurtingʼ	
	‘Even when you don't have a flare up it does not stop hurtingʼ	
Problem with daily activities and at-home productivity	‘Walking long distances - it hurtsʼ	15
	‘Can’t run, periodʼ	
	‘Can’t get up quicklyʼ	
	‘My gout is in my right hand so it hurts when I writeʼ	
	‘I have problems dressing myself because of the problems with swelling and painʼ	
	‘Affects things that I have to do around the houseʼ	
	‘I can’t walk when I get an attackʼ	
	‘Can’t do a lot of walkingʼ	
	‘Gout 3-4 times a year - and it hurts so bad I can't walk, or stand for the sheets to even touch itʼ	
	‘Sudden sharp pains in legs, thighs, and foot during and between attacksʼ	
	‘I had to limit my volunteeringʼ	
	‘Limits my going out of townʼ	
Stiffness in joints	‘I had stiffness in my right kneeʼ	11
Problem with shoes/footwear	‘Causes me to wear a certain type of shoes… definitely no open toe shoesʼ	8
	‘I cannot wear shoes at all when it flares upʼ	
	‘I go to church with house shoes onʼ	
	‘I used to love to wear heelsʼ	
Work productivity	‘I had to stop working - due to goutʼ	7
Diet changes	‘The food that I eat will make my gout flareʼ	6
	‘I have to be careful about what I eat even the medications that I takeʼ	
	‘Restrictive dietʼ	
	‘I won't eat anything when I go out somewhere because I am afraid the food might flare up my goutʼ	
Emotional impact	‘I cry a lot because it hurts really badly when I have an attackʼ	6
African-American women (n = 7)		
Problem with daily activities and at-home productivity and dependency	‘The way it affects my life is it affects my ability to walk around and move around all day. And due to the severity of swelling I'm not allowed to wear shoesʼ	26
	‘I can't go up and down the steps like I would likeʼ	
	‘Can't comb hairʼ	
	‘It affects me because I can't walk. When it flares up I can't walk at all. I will be in the bed for a while. I can't stand to put your foot on the pillowʼ	
	‘I could stand there and watch my feet start swellingʼ	
	‘Swelling of my hands makes it hard to open anything. I have to ask the neighbor for help sometimes. It’s hard to lift a skillet or any kind of pot or panʼ	
Problem with shoes/footwear	‘Hard to move and wear shoes on my feet no matter what kind or size. It's like it's hard to lift the shoe. I will be bed ridden can't get up and go	19
	‘Had to get support stockingsʼ	
	‘Most of the time I have to be barefootʼ	
	‘Mine flares up and I have a lot of pain. I can't wear flat shoes I have to wear a little wedge shoes so that my feet will be going down so I can walkʼ	
Gout flare pain	‘Very painful and it makes it hard to drive. Sometimes you can't drive at allʼ	13
	‘When mine flares in my foot I can't even let the sheet touch itʼ	
	‘When I am walking my feet will swell. Then my in-step starts aching like a toothache. It makes me cry. It's like you want to pop the joint, but it won't popʼ	
	‘Sharp pain in toeʼ	
	‘Loss of appetite during a flare. You end up losing weight because of thisʼ	
	‘You’re in so much pain you can't eatʼ	
My ability to sleep	‘My ability to walk stand and sleep. I toss from side to side. It keeps me up all the time day and night…especially during flaresʼ	13
Need for canes and crutches	‘Need for canes crutches and walkers -you feel so swollen you feel like you don't have any balance. I have fallen all over the placeʼ	8
Eating restrictions	‘Be choice-y about what I eat. I can't eat beef, red meat and seafood.	4
	‘You get tired of eating chicken all the timeʼ	
	‘Can’t drink alcohol especially beerʼ	
Work productivity	‘Causes problems with work. I had to stop working and I was put on disability.ʼ	4
	‘I was constantly on my feetʼ	
Crippling effect of flares	‘It cripples youʼ	4
	‘Can’t get out of the bed for a monthʼ	
	‘Can’t get to the bathroomʼ	
	‘Gout destroyed my finger ligamentsʼ	
Joint pain and stiffness	‘I have it all over my bodyʼ	4
	‘It’s in hips legs back of head armsʼ	
	‘(Aches and pains)… constant - all the timeʼ	
	‘Aches and pains and stiffness and numbness in joints all the timeʼ	
	‘Have to rock to get upʼ	
Caucasian women (n = 6)		
Unpredictability of flare that brings a normal day to a halt	‘Unpredictable and can strike at any time. It ruins any plans. I'm a liability to my family because they never know what to expectʼ	22
	‘Can’t make plans to do anything’	
	‘When I was having it so much. My daughter would call to ask me to babysit. It's like we both knew I couldn’t do it because we didn't know if when the time came I would be able to do itʼ	
	‘Makes life iffyʼ	
	‘You never know when it is going to comeʼ	
	‘Just walking though Wal-Mart and then you are limping to get outʼ	
	‘When the episodes are frequent in the feet I can't make plans because I can't be sure that I will be wellʼ	
Work productivity	‘When the gout is in the heel, I can't walk at all. So I can't get any work done or go anyplace. When it's in the toe I can walk but with pain difficult doing most things but I can do mostʼ	16
	‘I was not able to fix Easter dinnerʼ	
	‘Problem with Walking or exercise of any sort - It is hard to just walk through the house -I live alone and during a flare up I can't even get to the bathroomʼ	
	‘Activities that you would like to participate in. Ex. You can't walk so you more or less hit the sofa. This for me is exerciseʼ	
	‘It came out in the hand… So I could not writeʼ	
Dependency	‘I live alone… (am worried) when am I going to get the next flare upʼ	9
	‘My husband had to do it allʼ	
Medication side effects	‘The medication for gout causes other problems such as diarrhea, itching, difficulty breathingʼ	7
Modification in physical activity due to gout and to avoid flares	‘Must be careful the need to take extra cautionʼ	7
	‘Must be careful with activities and walking because any misstep, uneven pavement, large gravel can cause insult and precipitate goutʼ	
	‘If I didn't have gout I could walk like everyone elseʼ	
	‘You end up taking different routes than everyone elseʼ	
Problem with shoes/footwear	‘Shoes do not fit appropriately because of the foot bump from many attacksʼ	5
	**‘**I had to change my shoes. I can't wear fancy shoes anymoreʼ	
Deformity	My nail Bed is deformed. I have a deformity.	5
Pain interfering with sleep	‘It wakes me up. I want to sleep when I go to sleep but it hurts too badly to sleep. Can't touch the sheet or the bed to my footʼ	5
	‘You’ll be sound asleep and the pain will wake youʼ	
	‘I just put my foot off of the bedʼ	
	‘You twist the foot to get it in just the right position but nothing worksʼ	

*Since some concerns were ranked equally by a group and tied for a rank, some groups have more than seven concerns.

The top concerns/domains noted by the patients were mapped to the limitation of identity-relevant performances with a progressive loss of self, exemplified by the following themes.

#### Effect of gout flare on daily activities and the ability to do things

All ten groups listed this among their top concerns. Gout affected: (1) activities of daily living: walking, getting out of bed, going to bathroom, standing, climbing stairs, combing hair, cooking; (2) other instrumental activities: driving, writing, gardening, cleaning dishes; (3) the ability to take care of family; and (4) social activities such as going to church, traveling, going out of town.

#### Effect on work ability, and productivity and employability and at home productivity

Eight patient groups listed this among their top concerns. The ability to function normally at work, work productivity and relationships were affected. Several patients lost employment due to gout. Women reported decreased productivity at home and interference with their ability to take care of their families and the house.

#### Severe pain during a gout attack and its impact on physical and emotional health

This was among the top concerns in eight nominal groups. Patients had problem with physical (inability to get out of bed) and emotional health (crying due to pain) and systemic effects of severe gout pain such as the loss of appetite due to pain. All eight groups described the impact of gout on physical health. Five groups (four African American and one Caucasian) highlighted the impact of gout on emotional health.

#### Dependency on family and others

Five patient groups listed this among their top concerns. Patients reported needing help from family members during gout flares for household work (for example, with cooking), ambulation and for getting to the doctor’s office.

#### Difficulty with shoes

Four patient groups expressed difficulty in wearing shoes, going to church with home shoes on and the need to wear a wider shoe, shoes of a larger size or walk barefoot sometimes. Some patients had to stop wearing fancy shoes and heels.

#### Interference with sexual function

Four patient groups listed this among their top concerns (all male nominal groups). Patients described that gout flares affected their sexual function negatively, leading to problems in having sex as well as low/no desire to have sex, and gout affecting their marital relationship negatively.

#### Sleep disruptions

Four patient groups reported sleep problems due to gout, both an altered sleep pattern in general due to gout and severe disruptive effect of gout flares on sleep.

#### Bringing normal day to a halt and unpredictability

Two groups ranked this among their top concerns. Patients mentioned the need to stop doing everything when the flare hit. Unpredictability of gout flare was very concerning to patients. Patients said that gout flare ruined their plans and they were uncomfortable with the unpredictable aspect of gout flares.

#### Made life change in a lot of ways

One patient group pointed out that their life changed in a lot of ways due to gout. Another patient group highlighted that gout led to the lack of healthiness. One patient had a divorce due to his inability to do outdoor activities with his wife due to frequent gout flares. Patients had to accept a lot of things that they did not want to, and the experience with gout ‘…made them change the way they carried themselves out.ʼ

#### Effect on hobbies and social life

One patient group said that gout affected not only their ability to continue their hobbies including fishing, hunting, biking, riding motorcycles, playing music and running, but also with the ability to go to dinner, movies and church.

#### Seeking people like them in social circles

One patient group described that gout was shaping their social and work interactions and that it was becoming an integral part of their social interactions. Patients were seeking others with similar conditions and bonding with them.

Other concerns and domains were consistent with body failure, as described in the trajectory model, as noted below.

#### Joint swelling, tenderness, heat and stiffness

Six groups listed this among their top concerns. Patients reported joint swelling, heat and tenderness during flares, residual joint swelling after the flare and joint stiffness.

#### Food restrictions

Six groups listed this among their top concerns. Patients reported having to give up foods that they enjoyed (red meat, shellfish, seafood), the need to stay disciplined with food and alcohol, the uncertainty about exactly which foods to avoid and the discomfort with dietary changes that they had made and the fear of (specific) food items causing flaring-up of gout.

#### Medication-related issues

Six patient groups listed this among their top concerns. Patients reported the inconvenience of taking pills daily, the need for multiple medications, the side effects from medications and the concern of potential long-term side effects. In one group, three of the top seven concerns were related to the negative impact of medications on QOL.

#### Impact on other health conditions

Two patient groups were concerned about the impact of gout on their other health conditions, including hypertension, conflicting messages from doctors and their concern for drug-drug interactions.

### Gender differences in the impact of gout on lives of patients

All four female nominal groups and only one of the six male nominal groups mentioned problems with shoes. Similarly, two of the four nominal groups in women, but none in men, mentioned joint/limb deformity due to gout. Dependency concerns were mentioned by three of the four nominal groups in women, compared to two of the six nominal groups in men. Sex difficulties due to gout were mentioned in none of the groups in women and four of the six groups in men.

### Race differences in the impact of gout on lives of patients

Compared to Caucasians, more African American groups ranked the following among their top seven concerns: (1) dietary restrictions and difficulty with eating certain foods due to gout (red meat, shellfish, seafood, alcohol), 0/4 versus 6/6 groups; (2) severe/extreme pain, 0/4 versus 5/6 groups; (3) gout bringing the day to a ‘haltʼ, 0/4 versus 2/6 groups; (4) effect on emotional health/irritability, 1/4 versus 4/6 groups; and (4) the need to use canes/crutches during flares, 0/4 versus 2/6 groups. On the other hand, Caucasian groups were more likely to report the impact of gout on work productivity among their top seven concerns, 3/4 Caucasian groups versus 2/6 African American groups.

## Discussion

This is the first qualitative study to focus on the impact of gout on QOL in African Americans and women with gout, both understudied populations. An elegant previous study in 11 men with gout identified three themes - the lack of knowledge related to gout, the progressiveness of untreated gout and the impact of disease (pain, dependency on family members, isolation, work disability) [26]. The concept covered was broad and diverse and women were not included. Our study builds on this foundation [26] and adds significant knowledge to this area. Other qualitative and quantitative studies have covered an even wider range of gout-related issues including treatment, knowledge, disease monitoring, outcomes and patient and clinician perspective within each study [24,25,42]. The emergence of clinical trial and survey data showing QOL deficits in gout patients [18-23] indicates that we need a better understanding of the patient perspective of the impact of gout on their lives.

What does this study tell us that we did not already know? This is the first study to describe differences in QOL by gender and race in patients with gout. Compared to Caucasians, African Americans with gout were somewhat more likely to report dietary restrictions due to gout, associated emotional burden, severe pain during gout flares, the need for canes/crutches during flares and gout bringing their day to a halt. This indicates that the patient experience of gout may differ by race. Gout severity may be higher in African Americans, a hypothesis that needs to be tested in future studies. African Americans have a lower rate of use of urate-lowering drugs compared to Caucasians, which may indicate less optimal disease control in African-Americans with gout [14]. This may lead to higher disease burden and a greater impact on QOL.

Women were more concerned about the difficulty with footwear, dependency and joint deformity, while men were more concerned about interference with sexual activity. These gender differences might reflect different social roles and differing priorities. The somewhat higher mean age of women than men with gout (68 versus 63 years) may also have contributed to these differences. To our knowledge, this is the first study with an adequate number of minorities and women to allow such an analysis. Whether these findings also indicate a difference in disease manifestation by gender needs to be examined in future studies.

Our study highlights a major and profound impact of gout on patients’ QOL. Several aspects of impact of gout on patients’ lives have not been previously reported, including the effect of gout on sexual function, sleep, hobbies, employability, relationships etc. Patients recognized the impact of gout flares on the desire to have sex as well as problems having sex. Gout patients described sleep disruption, with problems falling asleep and gout flares waking them up. A recent clinical study showing an independent association of gout with sleep problems [43] and a pre-clinical study showing altered rapid eye movement sleep and total sleep time after an intra-articular injection of uric acid [44] are highly supportive. Sleep disturbances are common in rheumatoid arthritis [45,46], another inflammatory arthritis, and respond to treatment with disease-modifying agents that target cytokines [47]. It remains to be seen if effective treatment of gout can reduce sleep problems in gout patients.

Patients described a significant disruption of lifestyle due to gout. Patients experienced gout as a life-changing event leading to the loss of job, and some had to apply for disability. These are major life events and signify that the impact of this disease can be devastating in many patients. Patients also needed crutches and walking aids during acute gout flares and felt that they were aging prematurely. Our study confirmed the impact of gout on patients’ lives recognized in the earlier study [26], namely, severe pain, dependency, work disability, dietary restrictions and social isolation. These findings substantiate the disease burden and the broad impact on patients’ lives.

What should change in the clinical practice? These findings may not surprise rheumatologists. The impact of gout on patients’ lives may not be as apparent to primary care physicians, where most time during outpatient visits is spent discussing and managing other chronic co-existent conditions (for example, heart disease, hypertension), with little time left to assess the impact of gout. Our study indicates that due attention should be paid to gout-related symptoms and associated disability, due both to gout flares as well as chronic gout, given its profound effects on patients’ QOL.

What are the implications for research? Low rates of appropriate gout treatment [2,5-10], and considerable suffering attributable to gout [18-23], in conjunction with our study findings, imply that we need effective, novel interventions, to optimize gout management, for example, a nurse-led gout clinic [48]. Very few areas of medicine are as well-suited for rigorous translational research as gout.

Our findings were consistent with our theoretical model that proposes that an illness impacts a person when the BBC chain is destabilized. In particular, the themes/domains identified most commonly by the patients mapped to either identity-relevant performances with a loss of self or body failures; the most disruptive to the BBC chain ranked the highest. This finding also supports the utility of this model in a chronic musculoskeletal condition. Several aspects of impact of gout on patients’ lives (work productivity, job performance, social life) were similar to, but more severe than those described by Lambert *et al*. in patients with osteoarthritis [49].

The study findings must be interpreted considering the study strengths and limitations. Our study, purposefully oversampled for African Americans, is representative of patients with gout [50]. Our goal was to study an underserved and understudied population, that is, African Americans with gout. However, the majority of the patients were men, which is representative of this condition. Enough nominal groups were conducted within each strata of race and gender to achieve saturation, with a minor exception of Caucasian women, where only one nominal group could be conducted. This was due to the availability of very few Caucasian women with gout for study screening, which mirrors the epidemiology of gout in the US [1]. Another limitation was that disease characteristics (disease duration, flares, gout medications et cetera) and post-menopausal status were not obtained for the patients. This limits our ability to hypothesize whether the gender differences might be related to menopausal status. The study strengths include a community clinic-based sample; focus on a single question and inclusion of a large number of African Americans and women with gout.

## Conclusions

In conclusion, qualitative research assessing the impact of gout on patients’ lives and how this differs by gender and race was conducted. A significant impact of gout on patients’ lives above and beyond acute pain and functional limitations with gout flares was evident, including the impact on sexual function, sleep, social life, emotional health, hobbies and footwear. Most concerns expressed by patients mapped to our proposed trajectory model with the BBC chain. Several differences in the impact of gout on QOL by gender and race were noted. Future studies should explore the potential underlying mechanisms of these patient-identified QOL deficits and design interventions targeting these mechanisms.

The University of Alabama at Birmingham’s Institutional Review Board (IRB) approved this study and all investigations were conducted in conformity with ethical principles of research.

## Abbreviations

BBC: body, biographical time and conceptions of self; ICD-9-CM: International Classification of Diseases, ninth revision, common modification code; NGT: nominal group technique; QOL: quality of life; ULT: urate-lowering therapy.

## Competing interests

There are no competing conflicts/interest related to this study. JAS has received research and travel grants from Takeda and Savient, and consultant fees from Savient, Takeda, Regeneron and Allergan.

## Authors’ contributions

JAS designed the study, developed the protocol and obtained IRB approval, conducted the nominal sessions, analyzed the voting by the participants and the data, wrote the first draft of the manuscript and revised it and made the decision to submit it. All authors read and approved the final manuscript.
